# Meta-analysis data of the accuracy of tests for meat adulteration by real-time PCR

**DOI:** 10.1016/j.dib.2022.107972

**Published:** 2022-02-17

**Authors:** Aisha N. Iskakova, Gulyaim K. Abitayeva, Arman B. Abeev, Zinigul S. Sarmurzina

**Affiliations:** a Republican State Enterprise ``Republican Collection of Microorganisms'' of the Committee of Science of the Ministry of Education and Science of the Republic of Kazakhstan, Shokan Valikhanov Street, 13/1, Nur-Sultan 010000, Kazakhstan; b “ABIOTECH'', LLP, Shokan Valikhanov street, 13/1, Nur-Sultan 010000, Kazakhstan

**Keywords:** Adulteration, Real-time PCR, qPCR, Sensitivity, Specificity, Meta-analysis

## Abstract

Adulteration of meat products, including illegal substitution and addition of ingredients, tampering, and the misrepresentation and labelling of food or food ingredients, is becoming a more serious problem globally. The consequences of such manipulations can pose various health risks for consumers, including food allergies and poisoning. This study investigates the problem of meat product adulteration, and detection of the same using real-time polymerase chain reaction (qPCR).

Review question: What is the diagnostic accuracy of real-time PCR testing for the detection of meat adulteration?

A review via meta-analysis was conducted. Searches were conducted in the Web of Science and MEDLINE (February 2021). All data processing was carried out using Review Manager 5.4 and Meta-Disc 1.4 software.

## Specifications Table

**Table:** 

Subject	Biostatistics
Specific subject area	meat adulteration, diagnostic accuracy of the real-time PCR test, meta-analysis
Type of data	Table Figure
How the data were acquired	Systematic literature search and data extraction were conducted in Web of science and MEDLINE (February 2021).
Data format	Raw Analysed Filtered
Description of data collection	A systematic search was performed in the Web of science and MEDLINE databases up to February 2021. The search was carried out using the search terms: ((("meat"[MeSH Terms] OR "meat"[A*ll* F*ields*]) OR "poultry"[A*ll* F*ields*]) AND (pcr[All Fields] OR "polymerase chain reaction"[A*ll* F*ields*])) NOT "salmonella"[All Fields] NOT "virus"[All Fields] NOT "lactobacillus"[All Fields] NOT "bacteria"[All Fields] NOT "yeast"[All Fields] NOT "nematode"[All Fields] NOT "toxoplasma"[All Fields] NOT "Staphylococcus"[All Fields] NOT "metabolom"[All Fields] NOT "dietary"[All Fields] NOT "clostridium"[All Fields] NOT "feeding"[All Fields] NOT "disease"[All Fields] AND ((("meat"[MeSH Terms] OR "meat"[A*ll* F*ields*]) OR "poultry"[A*ll* F*ields*]) AND (pcr[All Fields] OR "polymerase chain reaction"[A*ll* F*ields*])) NOT "salmonella"[All Fields] NOT "virus"[All Fields] NOT "lactobacillus"[All Fields] NOT "bacteria"[All Fields] NOT "toxoplasma"[All Fields] NOT "Staphylococcus"[All Fields] NOT "metabolom"[All Fields] NOT "dietary"[All Fields] NOT "clostridium"[All Fields] NOT "feeding"[All Fields] NOT "disease"[All Fields] NOT "pseudomonas"[All Fields] NOT "listeria"[All Fields] NOT "campylobacter"[All Fields] NOT "transcriptome"[All Fields] NOT "Escherichia coli"[All Fields] NOT "carcass"[All Fields] NOT "infection"[All Fields] NOT "mycoplasma"[A*ll* F*ields*]. Studies were eligible for inclusion in the review if they evaluated the effectiveness of the Real-time PCR method for identifying meat products (poultry, beef, etc.) and compared with reference standards or methods. The publications were selected according to the following criteria: - Comparison results of PCR tests with the reference standards (samples or method) are available in studies; - The studies contain data on limit of detection, analytical sensitivity and specificity; - The study uses the real-time PCR method; - Studies published in English or Russian. Studies were excluded if the Сt value (cycle threshold for analytical specificity) and limit of detection was unavailable.
Data source location	Data was collected from Web of science and MEDLINE. The locations of the meat samples that qualified after applying the inclusion/exclusion criteria: - Shantou and Beijing, China; - Selandor and Kuala Lumpur, Malaysia; - The Netherlands; - Turkey.
Data accessibility	Data identification number: doi: 10.17632/33dr7pbxgp.1 Direct link: https://data.mendeley.com/datasets/33dr7pbxgp/1

## Value of the Data

• Food adulteration remains an important concern due to its impact on public health, economics, religious factors, effective control and regulation of proper labelling, as well as prevention of unfair competition between foreign and local producers. The adulteration of meat products is classified as a priority and is included in the category of frequently adulterated food products. This study investigated meat product adulteration by focusing on the detection of adulteration using real-time polymerase chain reaction (qPCR).
• Meat products are a staple part of the diet amongst the Kazakhstan population. In addition to local products, foreign producers sell their meat products in the Kazakhstan market. In this regard, the use of the results of the meta-analysis to assess the diagnostic accuracy of PCR tests for the detection of meat adulteration. The results will be useful in the development of protocols and generating regulatory documents presiding the stringency of meat screening requirements. Even though there are regulations and laws related to food safety in many countries, including Kazakhstan, information regarding the authentication of meat source (species) and purity is lacking. Further research is required to determine the degree of adulteration in the entire meat industry in Kazakhstan, which will provide the current specialised services of the Ministry of Health of the Republic of Kazakhstan with more complete data and regulatory frameworks.
• To conduct effective laboratory control, it is necessary to use modern, sensitive, and accurate analytical methods to detect species adulteration in food. These data will be used to make decisions related to quality control and the safety of meat products.

## 1 Data Description

**Fig. 1:**
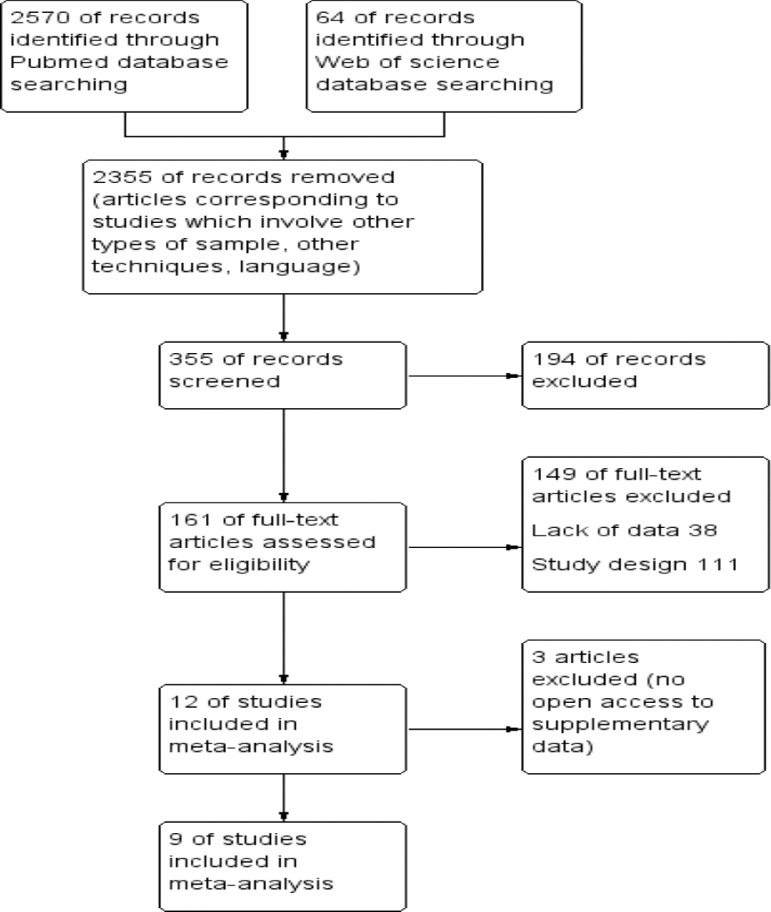
Flow diagram of included studies.

Fig. 1. A total of 2634 studies (2570 MEDLINE (PubMed) and 64 Web of Science, 09.02.2021) were found, of which, 336 studies were selected in PubMed and 19 in the Web of Science according to the selection criteria (2 355 articles were excluded during the screening phase). In total, 161 articles were selected for full text review after reviewing the abstract, 12 publications were selected for analysis, 3 more articles were excluded in the process of extracting data [1], [2], [3]. Finally, nine studies were selected for analysis, which fully met the selection criteria.

The exclusion criteria included disqualifying studies with an absence of the data required for analysis, the use of other/alternative methods of analysis, or modified versions of the qPCR. In addition, we excluded publications where the study objects (or meat source) were fish and marine animals.

Table 1. From the review process, we identified nine studies that fully met the selection criteria and were selected for the review. It should be noted that the study included those publications in which there was data based on the results of comparison with reference standards (samples or method). Most publications used the same quantitative PCR method but used primers on 16S or 18S rRNA. Thus, we monitored for the suitability of the obtained samples, the reagents used, and the course of the reaction itself.

**Table 1:** Characteristics of the included studies.

General study details					LOD			Specificity					
#	Authors	Target species	Method	Gene	Number of samples	Сt ± SD	Concentration [ng/μL]	Number of samples	Сt of target species	False-positive	True-positive	False-negative	True-negative
1a	Wang et al. [4]	horse	duplex qPCR	creatine kinase muscle (MCK)	90	36	0.01	21	22	0	3	0	18
1b	Wang et al. [4]	donkey	duplex RT PCR	creatine kinase muscle (MCK)	90	38	0.01	21	24	0	3	0	18
3	Li et al. [5]	mutton	qPCR	housekeeping gene replication protein A1 (RPA1)	18	29.91±0.00	0.5	6	26	0	1	0	5
74	Al-Kahtani et al. [6]	pork	qPCR	MericonTM Plant and Animal identification assays kit	6	32	0.001	42	16.4	0	6	0	36
149	Jonker et al. [7]	pork	qPCR	Cyt b gene, satellite IV	5	28.8	0.05	18	17.09	0	1	0	17
149a	Jonker et al. [7]	beef	qPCR	Cyt b gene, satellite IV	5	23.11	0.1	18	12.35	1	1	0	16
149b	Jonker et al. [7]	mutton	qPCR	Cyt b gene, satellite IV	5	32.1	0.05	18	20.12	0	1	0	17
149c	Jonker et al. [7]	horse	qPCR	Cyt b gene, satellite IV	5	35.6	0.05	18	21.02	0	1	0	17
149d	Jonker et al. [7]	chicken	qPCR	Cyt b gene, satellite IV	5	30.25	0.05	18	17.94	1	1	0	16
149e	Jonker et al. [7]	turkey	qPCR	Cyt b gene, satellite IV	5	28.63	0.05	18	17.9	1	1	0	16
130	Kesmen et al. [8]	chicken	qPCR	mitochondrial ND2	36	36.64±0.59	0.0001	42	17.52±0.34	0	6	0	36
130a	Kesmen et al. [8]	turkey	qPCR	mitochondrial ND2	36	37.82±0.41	0.0001	42	19.75±0.21	0	6	0	36
36	Ahmad Nizar et al. [9]	crocodile	duplex qPCR	Cyt b gene	25	30.65±0.25	0.004	45	17.36±0.2	0	3	0	42
40	Li et al. [10]	goat	qPCR	12S rRNA	NR	NR	NR	11	14	0	1	0	10
83	Rahman et al. [11]	dog	qPCR	Cyt b gene	NR	NR	NR	90	16.19±0.17	0	9	0	81
129	Ali et al. [12]	pork	qPCR	Cyt b gene	NR	NR	NR	99	15.48±0.14	0	9	0	90

qPCR – quantitative polymerase chain reaction, Cyt b – cytochrome b, NR – not reported, Ct *-* threshold cycle*,* SD – standard deviation.

The following data were extracted from the selected studies: title of the studies, names of the first author, year of publication, number of samples and species, methods, target gene, and test system results (Test results key: true positive = TP; true negative =TN; false positive = FP; false negative = FN; limit of detection = LOD; sensitivity; specificity) (Table 1). Data included here that was not provided in the main study was extracted from the supplementary material.

The specificity data of the qPCR reactions were extracted. For the target sample, the Ct level was obtained for 100% of the species type of the meat samples (mixes were not taken into account) and cross-reactivity with other types of animal and plant DNA was also conducted.

*Cytochrome b* gene was the most commonly used to detect the target species.

The limit of detection (LOD) was evaluated in targeted samples, the series of DNA dilutions of which was carried out only from pure targeted meat. DNA from mixes of different types of meat at a certain concentration and ratio were excluded from the calculation.

**Fig. 2:**
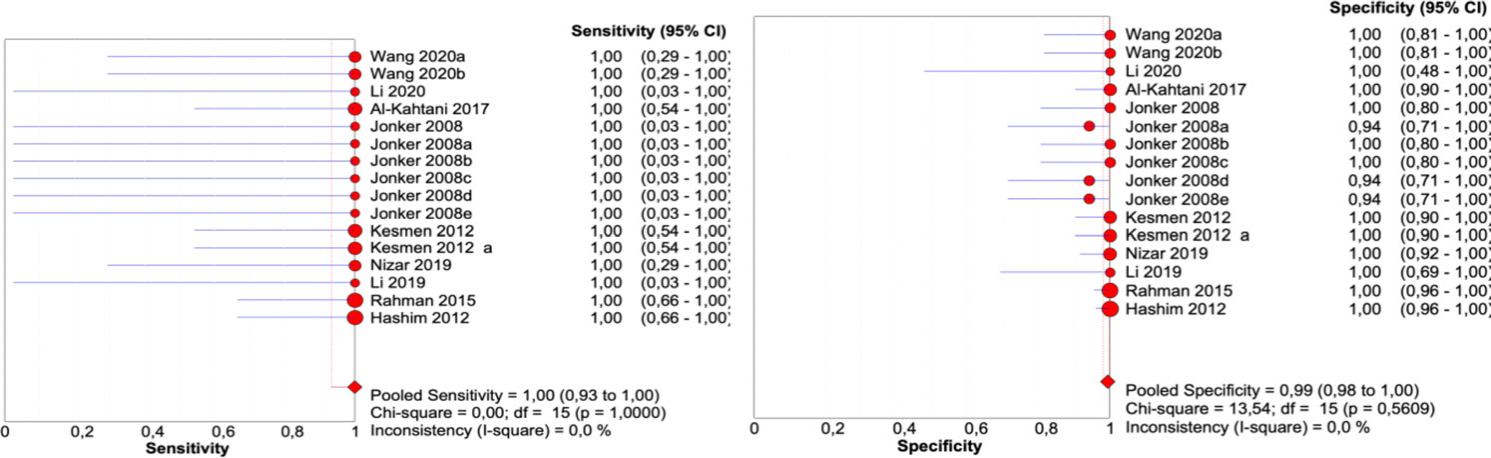
Results of sensitivity and specificity.

Fig. 2. Meta-analyses evaluating the reported test parameters for accuracy (including sensitivity and specificity) were conducted. Because there is no separate data on the number of false-positive, true-positive, false-negative, and true-negative results in many publications, the analysis used the results provided in the assessment of specificity. All data analyses were performed using Review Manager 5.4 software.

The sensitivity of the quantitative PCR method for identifying meat products when controlling for adulteration of products was 100%, 95% CI, 93.3%–100%; heterogeneity between trials of I^2^ = 0%. The results of specificity were 99.4%, 95% CI 98.2%–99.9%; heterogeneity between trials of I^2^ = 0%.

**Fig. 3:**
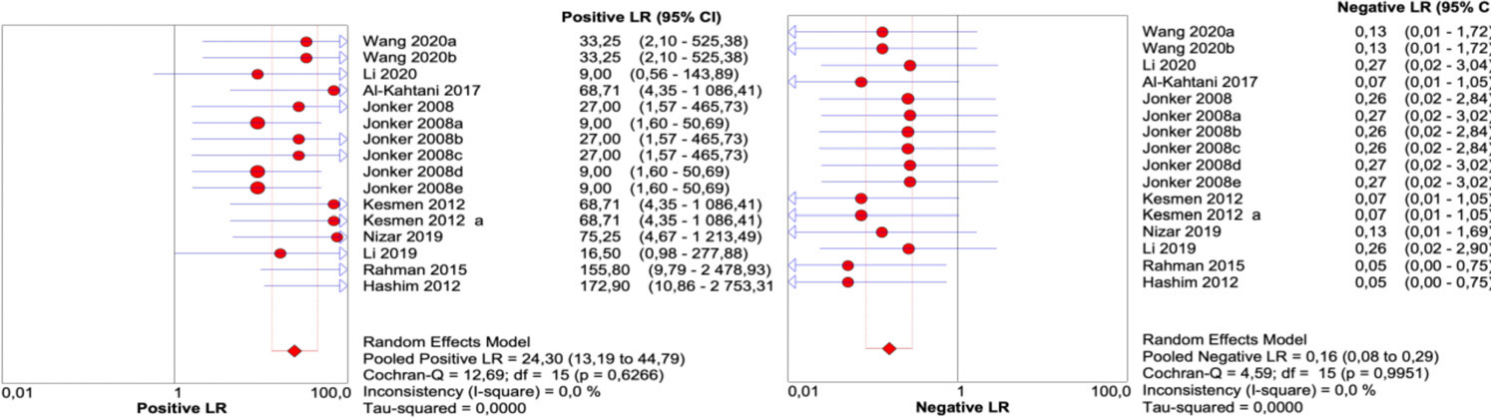
Results of the pooled positive likelihood ratio (PLR) and pooled negative likelihood ratio (NLR).

Fig. 3. Positive likelihood ratio (PLR) and negative likelihood ratio (NLR) were measured with a 95% confidence interval based on the TP, TN, FP, and FN rates that were extracted from the results of analytical specificity of included studies. The results of Pooled positive likelihood ratio (PLR) were 24.30, 95% CI, 13.19–44.79 and Pooled negative likelihood ratio (NLR) were 0.16, 95% CI, 0.08–0.29.

**Fig. 4:**
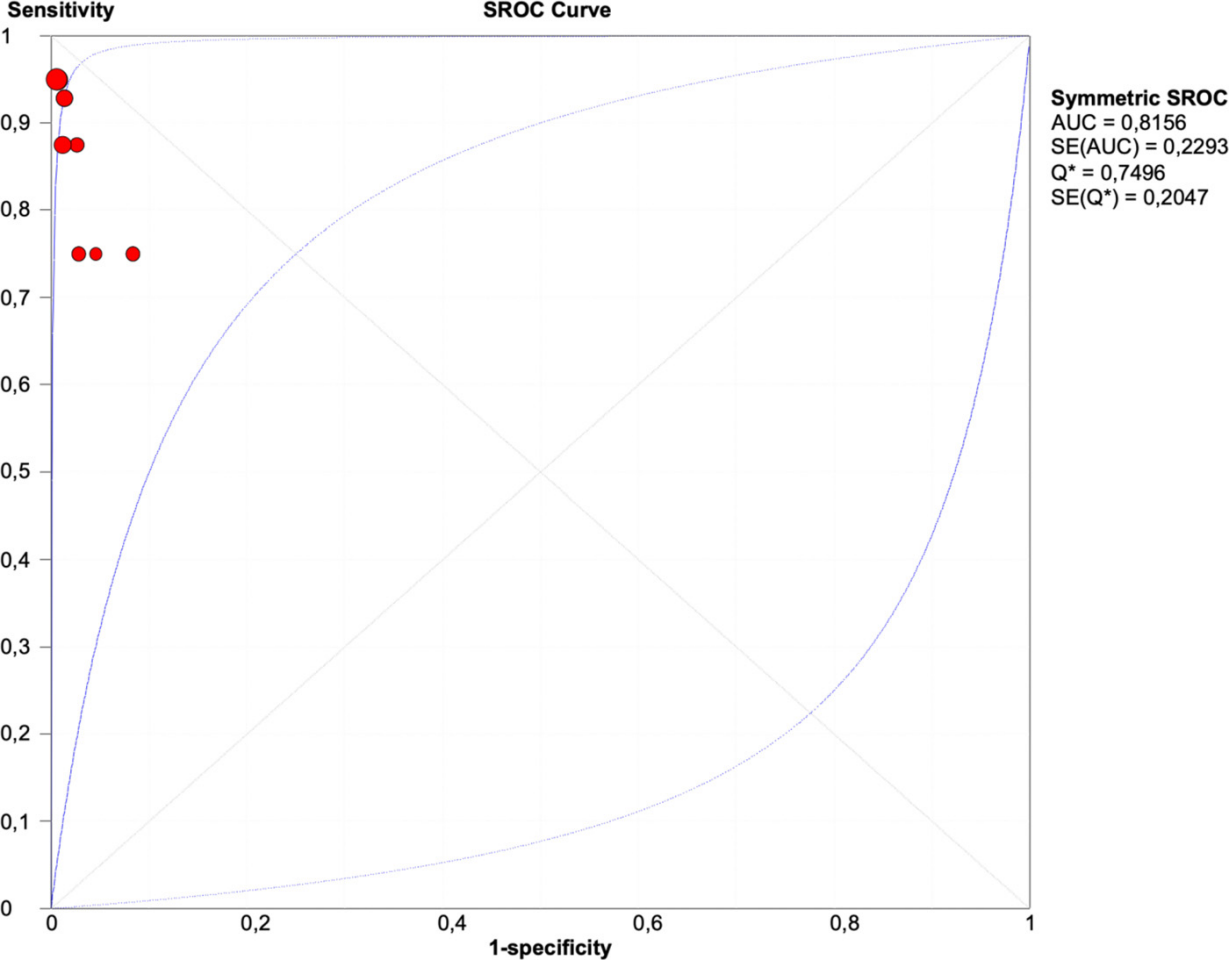
Results of sROC curve.

Fig. 4. Results of the sROC curve were performed using Meta-Disc 1.4 software. An area under the curve (AUC) close to 1 indicated a good diagnostic performance of the test. In this study the area under the curve was 81,56% (SE = 0.2293). A Q index greater than 0.5 (Q * = 0.7496) corresponds to the high efficiency of PCR tests for detecting falsified products.

## 2 Experimental Design, Materials and Methods

The meta-analysis results evaluating sensitivity indicate that controlling product adulteration is possible. We show that if the target species is present in all 100 samples, then all 100 adulterated products will be detected (that is, there are no false-negative samples). The specificity result of the meta-analysis suggests that if there are no target species in all 100 samples, then 0.6 samples will show an erroneous positive result (that is, there are false-positive samples) (Fig. 2).

This study was conducted according to the Preferred Reporting Items for Systematic Review and Meta-analysis of Diagnostic Test Accuracy Studies (PRISMA-DTA) statement [13].

*Search strategy and eligibility criteria.*

A systematic search was performed in the Web of Science and MEDLINE databases, including publications up to February 2021. The search was carried out using the search terms presented in «Description of data collection» section.

Studies were eligible for inclusion in the systematic review if they evaluated the effectiveness of the real-time PCR (qPCR) method for identifying meat products (poultry, beef, etc.) and compared with reference standards or methods. The publications were selected according to the following criteria:

- Comparison results of PCR tests with reference standards (samples or methods) available in the literature.
- The studies contain data on limit of detection, analytical sensitivity and specificity;
- The studies use the real-time PCR method;
- Studies published in English or Russian.

Studies were excluded if the Сt value (cycle threshold for analytical specificity) and the limit of detection were unavailable.

*Data extraction.* The research design of most studies on meat product adulteration is based on the use of prepared mixes with different meat concentrations. The presented data of the PCR test systems on real commercial samples of meat products are difficult to interpret as false positive, true positive, false negative, and true negative due to the lack of data on reference standards.

It should be understood that the results of reference standards, in this case, cannot be used in the classical sense of meta-analysis. For example, many publications use the same real-time PCR method as a standard method, but use primers for 16S rRNA [8,10] and 18S rRNA [9,11,12]; that is, positive results, when carrying out quantitative PCR, were evident in all analysed samples.

As a result of the aforementioned limitations, we decided to use the specificity analysis results. These results are the closest to those required for a meta-analysis to assess the diagnostic accuracy of the tests. These results are similar in all publications. In the selected studies, we can interpret the results as false positive, true positive, false negative, or true negative because we know the exact composition of the tested samples. In fact, the samples can be considered a standard.

Data extraction was conducted by one author (Iskakova, A.N.). The following data points were extracted from the selected studies: title of the studies, names of the first author, year of publication, number of samples and species, methods, target gene, and test system results (true positive, TP; true negative, TN; false positive, FP; false negative, FN; limit of detection, LOD; sensitivity; specificity) (Table 1). Data that were not provided in the main study are extracted from the supplementary material.

During the study of publications, some researchers used the analytical sensitivity concept as a synonym for the LOD concept. However, it is worth understanding that they are not interchangeable. The detection limit is the lowest detectable level of analyte distinguishable from zero. Whereas, the analytical sensitivity is the slope of the calibration curve. The analytical sensitivity indicates the capacity of the method to differentiate between two very close analyte concentrations [14].

The limit of detection (LOD) was evaluated in targeted samples, the series of DNA dilutions of which was carried out only from pure targeted meat. DNA from mixes of different types of meat at a certain concentration and ratio were not used in the calculation.

*Data analysis.* All data analysis were performed using Review Manager 5.4 and Meta-Disc 1.4 software. Sensitivity, specificity, positive likelihood ratio (PLR), and negative likelihood ratio (NLR) were measured with a 95% confidence interval based on the TP, TN, FP, and FN rates that were extracted from the results of analytical specificity of the included studies.

Sensitivity is the probability that a test result will be positive when the test target species exists (true positive rate) and calculated as  TP/(TP + FN).

Specificity is the probability that a test result will be negative when the test target species is not present (true negative rate) and calculated as  TN/(TN + FP).

SROC curves: An area under the curve (AUC) close to 1 indicated good diagnostic performance of the test.

Since we performed a meta-analysis of only one method (real-time PCR) and did not divide the data into subgroups, it was decided not to carry out the diagnostic odds ratio (DOR) analysis.

Quality assessment was not performed because the study was carried out for a meta-analysis, in which the results of a specificity test were used as data (that is, the samples themselves acted as a standard). In this regard, the given assessment results do not reflect the assessment of the entire study in publications, but only the data that were used for meta-analysis.

## Ethics Statement

Not applicable.

## CRediT authorship contribution statement

**Aisha N. Iskakova:** Methodology, Visualization, Formal analysis, Writing – original draft. **Gulyaim K. Abitayeva:** Project administration, Writing – review & editing. **Arman B. Abeev:** Conceptualization, Methodology, Validation. **Zinigul S. Sarmurzina:** Funding acquisition, Supervision, Resources.

## Declaration of Competing Interest

The authors declare that they have no known competing financial interests or personal relationships that could have influenced the work reported in this paper.

## Appendix Supplementary Materials

Image, application 1

Image, application 2
