# Supplementary material for: Meta-analysis data of the accuracy of tests for meat adulteration by real-time PCR
Source: Data Brief. 2022 Feb 17;41:107972. doi: 10.1016/j.dib.2022.107972 (PMC8881715; doi:10.1016/j.dib.2022.107972)
Supplement: Supplementary file 2 [file mmc2.zip › Tabular results - summary.rtf]

Summary Sensitivity

               Study     |	Sen	[95%  Conf. Iterval.]		TP/(TP+FN)  TN/(TN+FP)
--------------------------------------------------------------------------------------------
Wang 2020a               |	1,000     0,292   - 1,000   		 3/3	18/18
Wang 2020b               |	1,000     0,292   - 1,000   		 3/3	18/18
Li 2020                  |	1,000     0,025   - 1,000   		 1/1	5/5
Al-Kahtani 2017          |	1,000     0,541   - 1,000   		 6/6	36/36
Jonker 2008              |	1,000     0,025   - 1,000   		 1/1	17/17
Jonker 2008a             |	1,000     0,025   - 1,000   		 1/1	16/17
Jonker 2008b             |	1,000     0,025   - 1,000   		 1/1	17/17
Jonker 2008c             |	1,000     0,025   - 1,000   		 1/1	17/17
Jonker 2008d             |	1,000     0,025   - 1,000   		 1/1	16/17
Jonker 2008e             |	1,000     0,025   - 1,000   		 1/1	16/17
Kesmen 2012              |	1,000     0,541   - 1,000   		 6/6	36/36
Kesmen 2012
a           |	1,000     0,541   - 1,000   		 6/6	36/36
Nizar 2019               |	1,000     0,292   - 1,000   		 3/3	42/42
Li 2019                  |	1,000     0,025   - 1,000   		 1/1	10/10
Rahman 2015              |	1,000     0,664   - 1,000   		 9/9	81/81
Hashim 2012              |	1,000     0,664   - 1,000   		 9/9	90/90
--------------------------------------------------------------------------------------------
          Pooled Sen     |	1,000     0,933   - 1,000   
--------------------------------------------------------------------------------------------
Heterogeneity chi-squared = 0,00 (d.f.= 15) p = 1,000
Inconsistency (I-square) = 0,0 %
No. studies = 16.


Summary Specificity

               Study     |	Spe	[95%  Conf. Iterval.]		TP/(TP+FN)  TN/(TN+FP)
--------------------------------------------------------------------------------------------
Wang 2020a               |	1,000     0,815   - 1,000   		 3/3	18/18
Wang 2020b               |	1,000     0,815   - 1,000   		 3/3	18/18
Li 2020                  |	1,000     0,478   - 1,000   		 1/1	5/5
Al-Kahtani 2017          |	1,000     0,903   - 1,000   		 6/6	36/36
Jonker 2008              |	1,000     0,805   - 1,000   		 1/1	17/17
Jonker 2008a             |	0,941     0,713   - 0,999   		 1/1	16/17
Jonker 2008b             |	1,000     0,805   - 1,000   		 1/1	17/17
Jonker 2008c             |	1,000     0,805   - 1,000   		 1/1	17/17
Jonker 2008d             |	0,941     0,713   - 0,999   		 1/1	16/17
Jonker 2008e             |	0,941     0,713   - 0,999   		 1/1	16/17
Kesmen 2012              |	1,000     0,903   - 1,000   		 6/6	36/36
Kesmen 2012
a           |	1,000     0,903   - 1,000   		 6/6	36/36
Nizar 2019               |	1,000     0,916   - 1,000   		 3/3	42/42
Li 2019                  |	1,000     0,692   - 1,000   		 1/1	10/10
Rahman 2015              |	1,000     0,955   - 1,000   		 9/9	81/81
Hashim 2012              |	1,000     0,960   - 1,000   		 9/9	90/90
--------------------------------------------------------------------------------------------
          Pooled Spe     |	0,994     0,982   - 0,999   
--------------------------------------------------------------------------------------------
Heterogeneity chi-squared = 13,54 (d.f.= 15) p = 0,561
Inconsistency (I-square) = 0,0 %
No. studies = 16.


Summary Positive Likelihood Ratio (Random effects model)

               Study     |	LR+   	[95%  Conf. Iterval.]		% Weight
--------------------------------------------------------------------------------------------
Wang 2020a               |	33,250    2,104   - 525,38  		4,90
Wang 2020b               |	33,250    2,104   - 525,38  		4,90
Li 2020                  |	9,000     0,563   - 143,89  		4,86
Al-Kahtani 2017          |	68,714    4,346   - 1086,4  		4,90
Jonker 2008              |	27,000    1,565   - 465,73  		4,61
Jonker 2008a             |	9,000     1,598   - 50,691  		12,51
Jonker 2008b             |	27,000    1,565   - 465,73  		4,61
Jonker 2008c             |	27,000    1,565   - 465,73  		4,61
Jonker 2008d             |	9,000     1,598   - 50,691  		12,51
Jonker 2008e             |	9,000     1,598   - 50,691  		12,51
Kesmen 2012              |	68,714    4,346   - 1086,4  		4,90
Kesmen 2012
a           |	68,714    4,346   - 1086,4  		4,90
Nizar 2019               |	75,250    4,666   - 1213,5  		4,83
Li 2019                  |	16,500    0,980   - 277,88  		4,69
Rahman 2015              |	155,80    9,792   - 2478,9  		4,88
Hashim 2012              |	172,90    10,858  - 2753,3  		4,88
--------------------------------------------------------------------------------------------
 (REM) pooled LR+        |	24,303    13,188  - 44,786  
--------------------------------------------------------------------------------------------
Heterogeneity chi-squared = 12,69 (d.f.= 15) p = 0,627
Inconsistency (I-square) = 0,0 %
Estimate of between-study variance (Tau-squared) = 0,0000  
No. studies = 16.


Summary Negative Likelihood Ratio (Random effects model)

               Study     |	LR-   	[95%  Conf. Iterval.]		% Weight
--------------------------------------------------------------------------------------------
Wang 2020a               |	0,128     0,010   - 1,718   		5,89
Wang 2020b               |	0,128     0,010   - 1,718   		5,89
Li 2020                  |	0,273     0,024   - 3,044   		6,81
Al-Kahtani 2017          |	0,072     0,005   - 1,047   		5,55
Jonker 2008              |	0,257     0,023   - 2,839   		6,87
Jonker 2008a             |	0,273     0,025   - 3,020   		6,85
Jonker 2008b             |	0,257     0,023   - 2,839   		6,87
Jonker 2008c             |	0,257     0,023   - 2,839   		6,87
Jonker 2008d             |	0,273     0,025   - 3,020   		6,85
Jonker 2008e             |	0,273     0,025   - 3,020   		6,85
Kesmen 2012              |	0,072     0,005   - 1,047   		5,55
Kesmen 2012
a           |	0,072     0,005   - 1,047   		5,55
Nizar 2019               |	0,126     0,009   - 1,691   		5,89
Li 2019                  |	0,262     0,024   - 2,898   		6,86
Rahman 2015              |	0,050     0,003   - 0,750   		5,43
Hashim 2012              |	0,050     0,003   - 0,749   		5,43
--------------------------------------------------------------------------------------------
 (REM) pooled LR-        |	0,157     0,084   - 0,294   
--------------------------------------------------------------------------------------------
Heterogeneity chi-squared = 4,59 (d.f.= 15) p = 0,995
Inconsistency (I-square) = 0,0 %
Estimate of between-study variance (Tau-squared) = 0,0000  
No. studies = 16.
